# The Book World of Medicine and Science

**Published:** 1896-06-20

**Authors:** 


### THE BOOK WORLD OF MEDICINE AND SCIENCE.

CLINICAL DIAGNOSIS: A PRACTICAL MANUAL OF CHEMICAL AND MICROSCOPICAL METHODS. By W. G. AITCHISON ROBERTSON, M.D., F.R.C.P.E. (London: Scientific Press, 1896. Price 6s.)

Every year the mass of facts which have been accumulated in regard to chemical and microscopical methods of clinical diagnosis grows larger. So extensive in truth has our knowledge on this subject become that it is impossible to express it in literary form without filling volumes far too bulky to be readily referred to. Moreover, all this knowledge is far too complicated to be carried in the mind without running great risks of error in its practical application. Students and practictioners ought then to welcome heartily a handbook like the one before us, which contains the facts and nothing else, which without any pretence of literary veneer accurately and succinctly offers to one who is about to undertake the chemical or microscopical investigation of any specimen or any organ, just those points which he ought to know, describes exactly the methods he ought to pursue, mentions the things he ought to have at hand, and, if we may be allowed the expression, gives a recipe for the making of the investigation. Beginning with the nasal secretion, Dr. Aitchison Robertson gives details as to the character and examination of the sputum, the gastric secretion, vomited matter, poisons in vomit, the fæces, animal and vegetable parasites, the various discharges that are met with in disease, and of the blood and the urine. One great advantage of what one may call the "short method," the method of lists and tables rather than of wordy descriptions, is that so little space being used a little repetition is of no consequence; so that while under the heads of the various morbid conditions we find mention of the diseases in which they are mostly met with, we also find in each section, under the head of the different diseases, lists of the morbid conditions likely to be found in connexion with them. For quick reference this sort of double entry is very convenient. The book is quite up to date, and we think it is sure to be found extremely useful to the clinical observer if kept in its proper place, *i.e.*, lying in actual readiness by the side of the microscope and the test-tube stand.

DIRECTORY OF CHEMISTS AND DRUGGISTS. (Kelly and Co. Limited, High Holborn, W.C. Price 20s.)

We have received a copy of the eighth edition of this useful work of reference, in the pages of which will be found the names of over 40,000 persons connected with the drug and chemical trades, both wholesale and retail, in England, Scotland, and Wales, and in the principal towns in Ireland. It may be interesting to note in connection with the drug trade that the exports for the year 1894 amounted to over £9,000,000. This large figure makes no allowance for home consumption.

STREET'S LIST OF NEWSPAPERS, 1896. (5, Serle Street, W.C., and 30, Cornhill, E.C.)

A copy of this handsomely printed book, which contains an alphabetical list of newspapers, magazines, &c., published in Great Britain and Ireland, has been forwarded to us. The clearness of the type is equalled by the clearness and conciseness of the particulars given, and it will be found a most useful work of reference. The excellent maps accompanying the book show at a glance exactly how many papers there are for each district, and the areas they cover.

